# Uvarinol and Dichamanetin Derived from *Uvaria chamae* as Potential Dual-Site Inhibitors Against PBP2a in Methicillin Resistant *Staphylococcus aureus*: An In Silico Study

**DOI:** 10.3390/ph18040529

**Published:** 2025-04-04

**Authors:** Emmanuel Ayodeji Agbebi, Shalom Oluwafunke Adeyemi, Adetola Ibukunoluwa Adewale, Omolara Seun Ajofoyinbo, Ezekiel Abiola Olugbogi, Oluwatoyin Mary Oyinloye, Iyadunni Adesola Anuoluwa, Timothy Oluwaseyi Agbebi, Basiru Olaitan Ajiboye, Babatunji Emmanuel Oyinloye

**Affiliations:** 1 Institute for Drug Research and Development, S.E. Bogoro Center, Afe Babalola University, Ado-Ekiti 360001, Nigeria; shalomadeyemi07@gmail.com (S.O.A.); erioluwadaju.adetola@gmail.com (A.I.A.); 2 Department of Pharmacognosy and Natural Products, College of Pharmacy, Afe Babalola University, Ado-Ekiti 360001, Nigeria; 3 Department of Science and Laboratory Technology, Ekiti State University, Ado-Ekiti 362103, Nigeria; omolaraoluwaseun99@gmail.com; 4 Department of Biochemistry, School of Basic Medical Sciences, Babcock University, Ilishan-Remo 121003, Nigeria; olugbogiezekiel@gmail.com; 5 Department of Biological Sciences, College of Sciences, Afe Babalola University, Ado-Ekiti 360001, Nigeria; mooyinloye@abuad.edu.ng; 6 Department of Microbiology, Faculty of Science, University of Medical Sciences, Ondo 351101, Nigeria; ianuoluwa@unimed.edu.ng; 7 Department of Physiology, College of Medicine, University of Ibadan, Ibadan 200005, Nigeria; agbebitimothy2000@gmail.com; 8 Phytomedicine and Molecular Toxicology Research Laboratory, Department of Biochemistry, Federal University Oye-Ekiti, Oye-Ekiti 371104, Nigeria; basiru.ajiboye@fuoye.edu.ng; 9 Phytomedicine, Biochemical Toxicology and Biotechnology Research Laboratories, Department of Biochemistry, College of Sciences, Afe Babalola University, Ado-Ekiti 360001, Nigeria; 10 Biotechnology and Structural Biology (BSB) Group, Department of Biochemistry and Microbiology, University of Zululand, Kwa-Dlangezwa 3886, South Africa

**Keywords:** Methicillin-resistant *Staphylococcus aureus* (MRSA), penicillin-binding protein 2a (PBP2a), *Uvaria chamae*, Uvarinol, Dichamanetin

## Abstract

**Background/Objectives**: Methicillin-resistant *Staphylococcus aureus* (MRSA) is one of the resistant pathogenic microorganisms that poses a global health threat due to its resistance to β-lactam antibiotics where the protein penicillin-binding protein 2a (PBP2a) plays a crucial role in its resistance. This study explores the potential of phytochemicals from *Uvaria chamae*, a plant with known medicinal properties, to serve as dual-site inhibitors of PBP2a, targeting both the active and allosteric sites. **Methods**: Phytochemicals previously identified in *U. chamae* were subjected to molecular docking and molecular dynamics simulations to evaluate their binding affinities and stability at PBP2a’s active and allosteric sites. The compounds’ pharmacokinetic profiles were predicted in silico using SwissADME tools. Root-mean-square deviation (RMSD), radius of gyration, and binding free energy were analyzed for dynamic stability. **Results**: Among the evaluated compounds, Uvarinol and Dichamanetin demonstrated high binding affinities compared to the co-crystallized ligand and standard antibiotics like ceftaroline. Uvarinol exhibited the highest binding affinity at both sites, with a docking score of −14.94 kcal/mol and a predicted inhibition constant (Ki) of 0.01 nM. Molecular dynamics simulations further confirmed the robust stability of Uvarinol and Dichamanetin, as indicated by consistently lower RMSD values relative to the co-crystallized ligand. Pharmacokinetic predictions revealed favorable drug-likeness and low toxicity, although Uvarinol showed limited gastrointestinal absorption. **Conclusions**: Uvarinol and Dichamanetin show promise as dual-site PBP2a inhibitors, offering a novel strategy to combat MRSA resistance. Their structural and pharmacokinetic properties make them viable candidates for further development, though experimental validation and formulation optimization are necessary to overcome bioavailability challenges.

## 1. Introduction

Antimicrobial resistance (AMR) is an escalating global health concern that threatens the efficacy of existing antibiotics and undermines advances in medicine. It is estimated that by 2050, AMR could result in approximately 10 million deaths annually, making it a more pressing health crisis than cancer [1,2,3]. Among the most challenging resistant pathogens is methicillin-resistant *Staphylococcus aureus* (MRSA), a Gram-positive bacterium responsible for a wide array of infections ranging from skin and soft tissue infections to life-threatening conditions like sepsis and pneumonia. MRSA’s ability to resist the effects of almost all β-lactam antibiotics places a substantial burden on global healthcare systems, increasing treatment costs, hospitalization times, and mortality rates [4,5,6,7].

There are two major mechanisms that have been identified as responsible for the resistance of MRSA to β-lactam antibiotics; namely, inactivation of the antibiotic by β-lactamases and target bypass owing to the presence/acquisition of the *mecA* gene, which encodes the low-affinity penicillin-binding protein 2a (PBP2a). This protein is crucial for MRSA’s survival in the presence of β-lactam antibiotics as it facilitates transpeptidation during peptidoglycan synthesis, even when native PBPs are inhibited [8,9,10,11,12]. Structural studies have revealed that the active site of PBP2a is sterically restricted, preventing effective acylation by most β-lactams [10,13,14]. This steric hindrance, combined with the protein’s conformational flexibility, enables MRSA to withstand the action of antibiotics designed to disrupt cell wall synthesis. While new-generation β-lactams such as ceftaroline and ceftobiprole have shown improved binding to PBP2a, the rapid evolution of resistance necessitates alternative therapeutic strategies [13,15,16].

In addition to the active site, recent studies have identified an allosteric site in PBP2a that plays a critical role in its catalytic mechanism and resistance. The allosteric site, located approximately 60 Å from the active site, regulates the protein’s conformation. Binding of specific ligands at the allosteric site induces conformational changes that “unlock” the active site, increasing its accessibility and facilitating both antibiotic binding and peptidoglycan crosslinking [13,16]. For instance, ceftaroline’s dual action at the allosteric and active sites not only promotes acylation of PBP2a but also disrupts its function, highlighting the allosteric site as a viable target for drug design [15,16]. Exploiting this mechanism offers a promising strategy for overcoming MRSA resistance, especially when conventional approaches fail [13,17].

The ongoing search for novel antimicrobial agents has revived interest in natural products, which have historically served as an invaluable source of therapeutic compounds. Plants, in particular, have yielded numerous clinically significant antibiotics and remain a rich reservoir of bioactive molecules [18,19,20]. Natural products have accounted for a significant proportion of the antibiotics in clinical use today, with their structural diversity offering a foundation for the discovery of new drugs [21]. Among these, the *Uvaria* genus of the Annonaceae family has gained attention for its pharmacological properties, including antimicrobial activity [22]. One notable species, *Uvaria chamae*, commonly referred to as “finger root” or “bush banana”, is a climbing shrub native to the tropical forests of West and Central Africa. Traditionally, *U. chamae* has been used to treat various ailments such as infections, bronchitis, wounds, and gastrointestinal disorders [23,24]. Phytochemical analyses of *U. chamae* have identified a diverse array of bioactive compounds, including flavonoids, alkaloids, acetogenins, and essential oils, which are believed to contribute to its medicinal properties [24,25,26,27].

Recent research has underscored the role of secondary metabolites in plants as potential modulators of bacterial resistance mechanisms. For instance, flavonoids and alkaloids have been reported to disrupt bacterial cell wall synthesis or inhibit resistance enzymes [8,28]. Additionally, essential oils and acetogenins have been shown to interfere with bacterial quorum sensing and membrane integrity [27,29]. These findings suggest that *U. chamae* may harbor compounds capable of targeting both the active and allosteric sites of PBP2a, thereby overcoming MRSA resistance.

Despite its traditional usage, there is limited understanding of the specific phytochemicals in *U. chamae* responsible for its antimicrobial effects, particularly against MRSA. Moreover, the potential interactions of these phytochemicals with critical resistance determinants like PBP2a remain unexplored. Targeting both the active and allosteric sites of PBP2a using natural products could provide a novel approach to combating MRSA infections. Investigating the mechanisms through which *U. chamae* phytochemicals inhibit PBP2a can bridge the gap between traditional medicinal knowledge and modern pharmaceutical research.

This study aims to identify phytochemicals from *U. chamae* with potential anti-MRSA activity, focusing on their interactions with both the active and allosteric sites of PBP2a. By employing molecular docking, dynamic simulations, and pharmacokinetic profile prediction, we seek to elucidate the binding mechanisms of these compounds and assess their efficacy in overcoming MRSA resistance. Understanding these interactions may pave the way for the development of novel antimicrobial agents, offering an alternative strategy to manage MRSA infections and mitigate the growing threat of antimicrobial resistance.

## 2. Results

### 2.1. Molecular Docking

**Figure 1:**
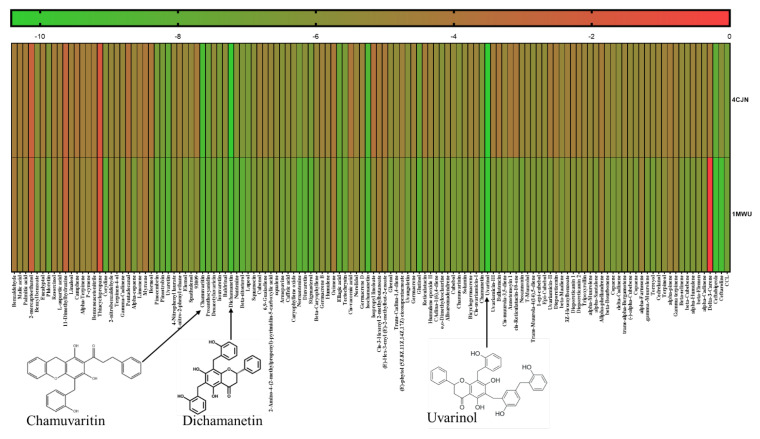
A heatmap showing the docking scores of the phytochemicals against PBP2a allosteric site (4CJN) and active site (1MWU). CCL-Co-crystallized ligand.

The study explored the antimicrobial potential of phytochemicals from *U. chamae* against MRSA, focusing on their interactions with PBP2a at its allosteric (PDB ID: 4CJN) and active sites (PDB ID: 1MWU). Two lead phytochemicals (Uvarinol and Dichamanetin) were identified based on molecular docking, dynamic simulations, and in silico pharmacokinetics. The docking study revealed that Uvarinol, Dichamanetin, and Chamuvaritin exhibited higher binding affinities at both the allosteric (PDB ID: 4CJN) and active sites (PDB ID: 1MWU) of PBP2a compared to co-crystallized ligands and standard antibiotics (Ceftaroline and Ceftobiprole), as shown in Figure 1 and Table 1 below. Figure 1 is a graphical representation showing the docking scores of all the phytochemicals in *U. chamae*, the standard antibiotics (Ceftaroline and Ceftobiprole), and the co-crystallized ligand.

**Figure 2:**
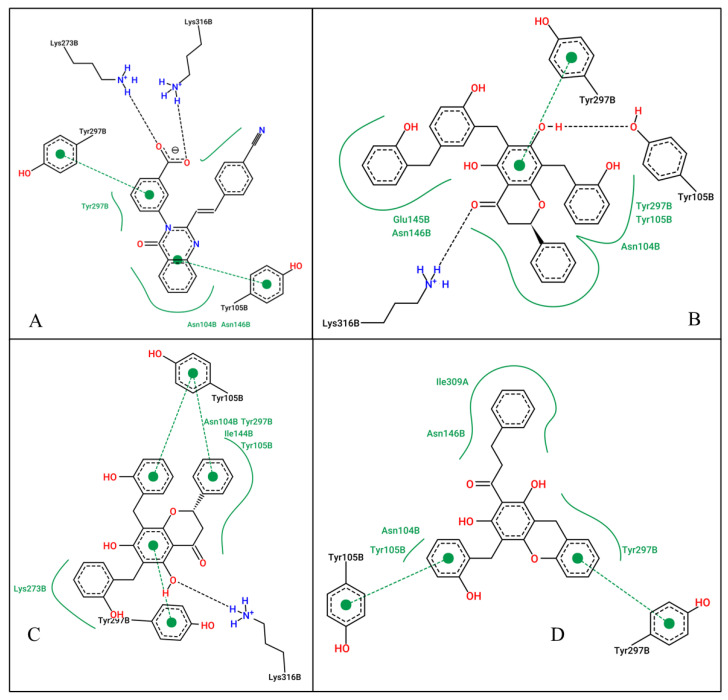
2D ligand interactions of the co-crystallized ligand (**A**), Uvarinol (**B**), Dichamanetin (**C**), and Chamuvaritin (**D**) at the allosteric site of the PBP2a receptor.

**Figure 3:**
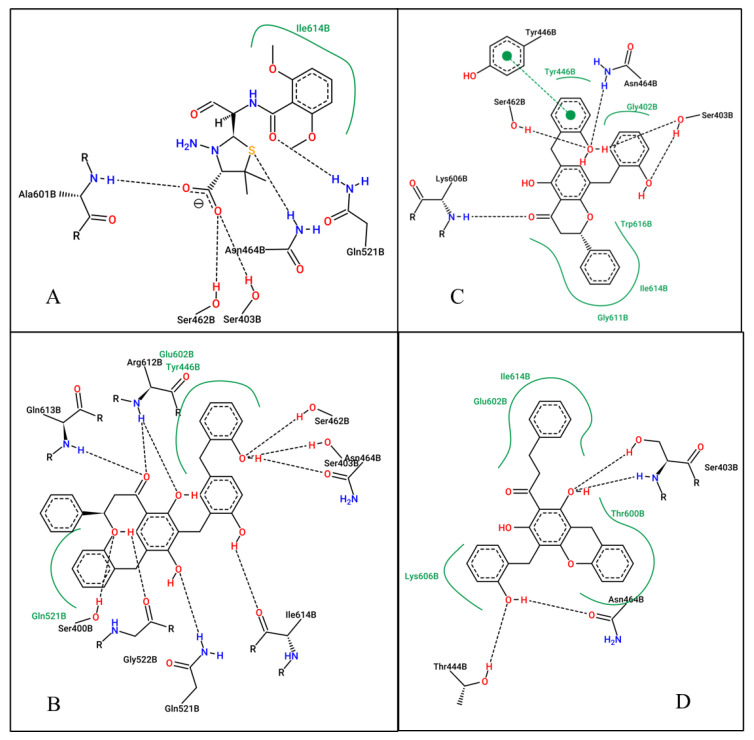
2D ligand interactions of the co-crystallized ligand (**A**), Uvarinol (**B**), Dichamanetin (**C**), and Chamuvaritin (**D**) at the active site of the PBP2A receptor.

Since these compounds have higher binding energy than the respective co-crystallized ligands and standard drugs (Ceftaroline and Ceftobiprole), they were selected as the hit compounds to be used for further studies. Table 1 summarizes their docking scores, predicted inhibition constants (Ki), and interacting residues, while Figure 2 and Figure 3 shows the 2D ligand-protein interaction diagram of these compounds.

**At the allosteric site (4CJN):** Uvarinol showed the highest binding affinity (docking score: −10.42 kcal/mol, Ki: 22.89 nM), followed by Dichamanetin (−10.32 kcal/mol, Ki: 27.07 nM). Comparatively, CCL (QNZ) and Chamuvaritin showed weaker binding affinities, with docking scores of −9.16 and −9.17, and Ki values of 192.16 nM and 189.39 nM, respectively. The superior binding of Uvarinol was attributed to its extensive interactions, including hydrogen bonds with LYS316 and TYR105, hydrophobic interactions with residues such as ASN104, TYR105, GLU154, ASN146, and TYR297, and π-π stacking interactions with TYR297. Dichamanetin also formed strong interactions, notably hydrogen bonding with LYS316, hydrophobic contacts with multiple residues, and π-π stacking with TYR105 and TYR297. In contrast, CCL and Chamuvaritin formed fewer interactions, which likely contributed to their weaker binding affinities.

**At the active site (1MWU):** Uvarinol once again emerged as the top-performing compound with an exceptionally low docking score of −14.94 and a Ki of 0.01 nM, indicating extremely high potency. Dichamanetin also demonstrated strong binding, with a docking score of −12.01 and a Ki of 1.57 nM. CCL (7EP) and Chamuvaritin exhibited moderate binding affinities, with docking scores of −8.49 and −11.25, and Ki values of 601.23 nM and 5.66 nM, respectively. Uvarinol and Dichamanetin also exhibited superior binding affinity compared to standard antibiotics (Ceftobiprole: docking score: −10.65 kcal/mol, Ki: 15.57 nM; and Ceftaroline: docking score: −10.32 kcal/mol, Ki: 27.28 nM). The superior performance of Uvarinol was due to its ability to form numerous interactions, including hydrogen bonds with residues such as SER400, SER403, SER462, ASN464, GLN521, GLY522, ARG612, GLN613, and ILE614, as well as hydrophobic contacts with TYR446, GLN521, and GLU602. Dichamanetin also formed multiple hydrogen bonds with residues including SER403, TYR446, and ASN464, and hydrophobic interactions with residues like GLY402, ILE614, and TRP616. In contrast, the interactions formed by CCL and Chamuvaritin were less extensive, resulting in weaker binding. Overall, Uvarinol consistently demonstrated the strongest binding affinity and potency against both 4CJN and 1MWU targets. The compound’s ability to form numerous hydrogen bonds, hydrophobic interactions, and π-π stacking interactions underscores its potential as a lead compound. Dichamanetin also showed promising results, making it a strong candidate for further investigation. Therefore, the dynamic behaviors/interaction of these compounds—Uvarinol and Dichamanetin were investigated using the molecular dynamic (MD) simulation.

### 2.2. Validation of Docking Protocol

**Figure 4:**
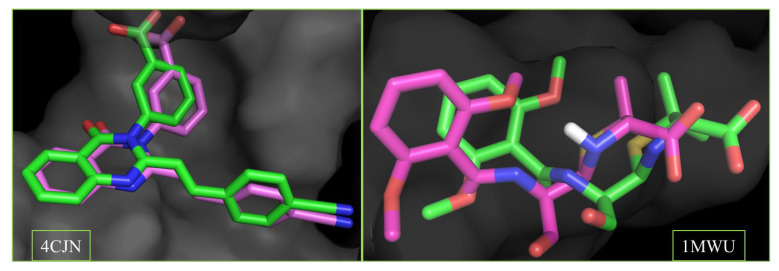
Superimposed structures of the co-crystalized ligands in their co-crystallized (green) and re-docked poses (magenta) at the allosteric (4CJN) and active site (1MWU) of the receptor. (RMSD = 0.146 and 0.968 A, respectively). Color code: carbon (green and magenta), hydrogen (white), nitrogen (blue) and oxygen (red).

The accuracy of the docking procedure was validated by re-docking co-crystallized ligands into the receptor’s binding sites. The low root-mean-square deviation (RMSD) values of 0.146 Å (4CJN) and 0.968 Å (1MWU) confirmed the reliability of the docking protocol. Superimposed ligand structures are shown in Figure 4.

### 2.3. Molecular Dynamics (MD) Simulation

**Figure 5:**
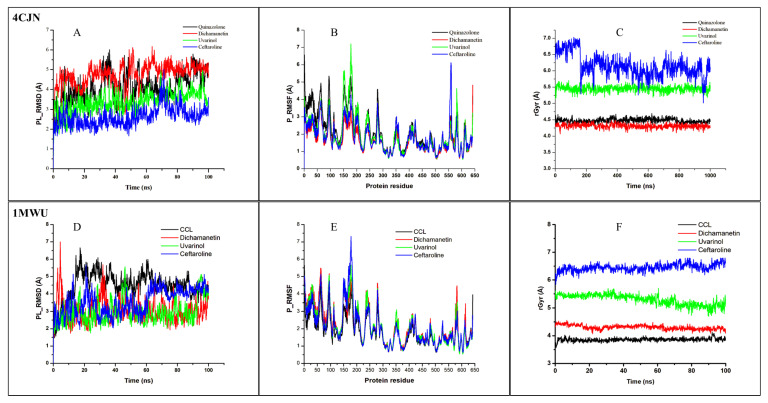
MD simulation results of 4CJN (allosteric site) and 1MWU (active site) complexed to their respective co-crystallized ligands (CCL), Uvarinol, Dichamanetin, and Ceftaroline. (**A**,**D**) RMSD, (**B**,**E**) RMSF, (**C**,**F**) rGyr, graphical plot. All simulations were carried out using Schrödinger’s Maestro suite (Desmond).

Molecular dynamic (MD) simulations were conducted to evaluate the dynamic stability, structural, and functional relationship of the PBP2a-ligand complexes. MD simulation mimics the biological system and provides information about the complex stability, conformational changes, and the flexibility/fluctuation of each protein residue during simulation [30]. Key parameters, such as the root mean square deviation (RMSD), root mean square fluctuation (RMSF), and radius of gyration (rGyr), were analyzed (Figure 5 and Table 2).

### 2.4. At the Allosteric Site (4CJN)

The RMSD provides information about the degree of stability and structural/conformational variation in a ligand–protein complex over time [30,31]. A consistent and low RMSD value shows that the ligand maintains a similar pose to its docking/starting pose during the simulation, indicating stability, while a fluctuating RMSD value shows that there is a frequent alteration in the ligand pose in the binding pocket, indicating instability [32]. From our results (Table 2, Figure 5), it was observed that Uvarinol has a lower RMSD value compared to the co-crystallized ligand, QNZ (3.386 ± 0.016 Å vs. 4.083 ± 0.024 Å, respectively) while maintaining a steadier/more consistent value throughout the 100 ns simulation, indicating its stability at the receptor site. Dichamanetin has a relatively higher RMSD value of 4.752 ± 0.018 Å, while Ceftaroline has the least RMSD value of 2.625 ± 0.015 Å. This result supports our findings from the molecular docking study, where Uvarinol showed very high binding affinity and potentials compared to Dichamanetin and the co-crystalized ligand (Quinazolinone).

The RMSF values and plots reveal the dynamics/movement of the protein residues, and the key residues involved in the strongest interactions with the ligands throughout the 100 ns simulation. The compounds had similar RMSF to the co-crystallized ligand and maintained a low RMSF value at the most important residues (Figure 5). This indicates stable ligand binding, evidenced by the minimal movements in the binding region.

The rGyr is a measure of the protein stability and compactness. A lower rGyr values indicate the protein’s stability and compactness during the simulation period and vice versa. Dichamanetin has a similar value to the CCL (4.312 Å ± 0.002 vs. 4.478 ± 0.002 Å) while Uvarinol has an average rGyr value of 5.452 ± 0.002 Å. Their plots revealed that they were stable throughout the simulation. This provides more evidence to support our molecular docking and RMSD findings that Uvarinol and Dichamanetin may be effective allosteric modulator of the PBP2a enzyme activity.

### 2.5. At the Active Site (1MWU)

The results indicated that Uvarinol and Dichamanetin exhibited lower RMSD values compared to the co-crystallized ligand, 7EP (2.955 ± 0.022 Å, 3.036 ± 0.021 Å, and 4.448 ± 0.027 Å, respectively). Notably, the RMSD value of Dichamanetin is comparable to that of the standard drug, Ceftaroline (3.036 ± 0.021 Å vs. 3.550 ± 0.023 Å), suggesting similar binding stability. These findings suggest strong binding affinity at the protein’s active site, consistent with molecular docking predictions.

Regarding structural flexibility, the co-crystallized ligand 7EP exhibited the lowest RMSF value (1.809 ± 0.030 Å), while Dichamanetin showed the highest (2.139 ± 0.039 Å). Interestingly, the RMSF values of Uvarinol and Ceftaroline were nearly identical (2.052 ± 0.039 Å and 2.030 ± 0.040 Å, respectively), indicating comparable dynamic fluctuations during the simulation.

The radius of gyration (rGyr) values reveals notable differences among the ligands that can be interpreted in terms of compactness and conformational behavior. The co-crystallized ligand, 7EP (CCL), has the smallest rGyr (3.859 ± 0.002 Å), suggesting that it adopts a highly compact and potentially optimal binding pose within the active site of PBP2a. In contrast, Dichamanetin showed a slightly larger rGyr (4.303 ± 0.002 Å), which may indicate minor conformational adjustments upon binding while still maintaining a relatively tight complex. Moving further, Uvarinol and Ceftaroline exhibited even higher rGyr values (5.294 ± 0.006 Å and 6.450 ± 0.004 Å, respectively), pointing toward more extended conformations or increased molecular flexibility. This extended state might either facilitate a broader interaction surface with the receptor or, alternatively, suggest a less compact fit compared to 7EP, potentially influencing the stability of the complex. Overall, these differences in rGyr underscore the varied binding modes and structural adaptations of the ligands, with the lower rGyr of 7EP serving as a reference for an ideal, compact binding configuration, while the higher values for Uvarinol and Ceftaroline hint at more dynamic or flexible interactions at the receptor’s active site.

The data from the molecular dynamic simulations revealed that Uvarinol and Dichamanetin were relatively more stable than the co-crystalised ligand and standard drug, Ceftaroline. These findings support their potential as effective modulators of PBP2a enzyme activity, warranting further investigation into their therapeutic relevance.

### 2.6. MM/GBSA Binding Free Energy

For the 4CJN complex, Uvarinol exhibited a more favorable binding energy (–44.30 kcal/mol) than Quinazolinone and Dichamanetin, aligning with its superior docking score, lower RMSD, and stability during simulation. However, Ceftaroline shows the best binding energy (–65.82 kcal/mol), outperforming all compounds. Similarly, in the 1MWU complex, while Uvarinol demonstrates improved binding over 7EP (CCL) and Dichamanetin, Ceftaroline again achieved the most favorable binding energy, indicating its stronger interaction with the protein, as shown in Table 3 below. Overall, Uvarinol performed well in terms of docking and stability while Ceftaroline consistently achieved the strongest binding energy.

### 2.7. Pharmacokinetic Predictions

In silico pharmacokinetic profiling of the hit compounds and standards were performed using SwissADME. The result revealed favorable absorption, distribution, metabolism, excretion, and toxicity (ADMET) properties for Dichamanetin and Uvarinol. For drug-likeness, while Dichamanetin showed no violations, Uvarinol exhibited one Lipinski rule violation due to its higher molecular weight (574.62 g/mol), but its other properties, including hydrogen bond acceptors (7) and donors (5), were within acceptable limits, as shown in Table 4. Generally, synthetic accessibility, which predicts the ease of synthesizing the chemical compound, ranges from 1 to 10, indicating very easy to synthesize to very difficult to synthesize. This means that, the lower the value, the easier it is to be synthesized; therefore, a lower value is desirable. Dichamanetin and Uvarinol have synthetic accessibility scores of 4.15 and 4.70, respectively, indicating moderate ease of synthesis.

For bioavailability, it ranges from 0 to 1, indicating not bioavailable to 100% bioavailable. Usually, any compound with a bioavailability score of ≥0.55 is considered ideal and absorbed very well by the body. Uvarinol had a bioavailability score of 0.55, similar to Dichamanetin, although its gastrointestinal absorption was predicted to be low, potentially limiting its oral bioavailability. Therefore, these compounds have favorable/acceptable pharmacokinetic and drug-likeness profiles. For the toxicity profile, as shown in Table 5, Uvarinol is not an inhibitor of key CYP450 enzymes, while Dichamanetin is a potential inhibitor of CYP2C19 and CYP2C9, indicating a low likelihood of metabolic interactions.

## 3. Discussion

Studies on natural product dual-site inhibitors of PBP2a are limited, but previous research has identified flavonoids and alkaloids as effective inhibitors of bacterial resistance mechanisms, including PBP2a [8,28]. The findings of this study highlight the potential of phytochemicals from *Uvaria chamae,* specifically Uvarinol and Dichamanetin, as dual-site inhibitors of PBP2a, a critical enzyme in the resistance mechanism MRSA. By engaging both the active and allosteric sites of PBP2a, these compounds mirror the mechanism of clinically approved agents like ceftaroline, which induces conformational changes that enhance active site accessibility and disrupt cell wall synthesis [15,16,33].

Mechanistically, Uvarinol and Dichamanetin interact with key residues such as Lys273, Lys316, Tyr105, and Gln521, akin to known inhibitors. Quinazolinone {(E)-3-(3-carboxyphenyl)-2-(4-cyanostyryl) quinazolin-4(3H)-one}, for example, has been known to form hydrogen bonds with Lys316 and Lys273 by extending into the pocket formed by the amino acids Lys316, Lys273, and Glu294 at the allosteric site [15,34]. This form of interaction was also observed for Uvarinol and Dichamanetin at the allosteric site (as shown in Figure 2). Binding free energy analysis further supports their potential: Uvarinol exhibited the strongest binding affinity (−44.30 ± 0.42 kcal/mol), closely followed by Dichamanetin (−43.48 ± 0.50 kcal/mol), both outperforming the co-crystallized ligand QNZ (−37.25 ± 0.76 kcal/mol). These findings suggest that both Uvarinol and Dichamanetin form energetically favorable and stable complexes with PBP2a, compared to the co-crystalized inhibitor, QNZ. Uvarinol’s superior binding affinity can be attributed to its large molecular surface area (MolSA: 496.6 ± 0.13 Å^2^) and robust polar interactions, as reflected in its high polar surface area (PSA: 164.8 ± 0.18 Å^2^). Despite its relatively high radius of gyration (rGyr: 5.452 ± 0.002 Å) and solvent-accessible surface area (SASA: 444.4 ± 1.172 Å^2^), which indicate a slightly expanded and more solvent-exposed binding mode, Uvarinol compensates with its extensive interactions with the receptor. This suggests that Uvarinol’s binding strategy involves leveraging its structural flexibility and large interaction surface to form a stable complex with PBP2a. In contrast, Dichamanetin adopts a more compact configuration (rGyr: 4.312 ± 0.002 Å; SASA: 224.2 ± 0.94 Å^2^) that favors a tightly bound and stable complex, as reflected by its low RMSF values.

These differences in binding strategies underscore the complementary advantages of the two compounds, which could be exploited in future drug design efforts. Uvarinol’s extensive interaction surface and structural flexibility may allow it to adapt to and engage a broader range of residues, while Dichamanetin’s compactness ensures a robust, stable fit within the binding pocket. Both binding modes contribute to overcoming resistance by enhancing non-covalent interactions and their unique structures makes them non-susceptible to β-lactamase activity.

A recent study by [35] identified Quercetin and Daidzein as allosteric modifiers of MRSA’s PBP2a for restoring antibiotic effectiveness. These compounds have similar backbone/core structures with Uvarinol and Dichamanetin. In addition, Uvarinol and Dichamanetin, unlike Quercetin and Daidzein, have additional substituents (e.g., methoxy groups and aromatic chains) that interact with hydrophobic residues within the binding site. The additional hydrophobic features in Uvarinol and Dichamanetin improved their binding affinity and enhanced stability by engaging additional hydrophobic contacts, making them potentially more effective inhibitors. The planar geometries of Quercetin and Daidzein were reported to be crucial for modulating the conformational dynamics of PBP2a [35]. In the same manner, the overall planar structure of these compounds allows them to fit snugly within the hydrophobic allosteric site of PBP2a. Thus, the aromatic cores, hydroxyl groups, and planar geometries of these compounds help them to effectively bind and inhibit PBP2a’s allosteric site.

At the active site, previous studies have identified the key residues contributing to transpeptidation reaction [10]. Key active site residues identified as crucial for the interaction with β-lactam antibiotics include Ser403 (functions as the nucleophile in the transpeptidation reaction), Asn464 (stabilizes the transition state during the acylation process), and Tyr446 (contribute to the formation of a hydrophobic pocket that accommodates the β-lactam ring) [10,36,37]. Our compounds, Uvarinol and Dichamanetin, interact with these residues in the same manner as the known inhibitors. In addition, [10] suggested that increasing the non-covalent interaction will improve the binding affinity at the active site. This was observed for our compounds where more non-covalent interaction led to improved binding affinity, as shown in Table 1 and Figure 3. The numerous hydrophobic and hydrogen bonds formed by these compounds will enhance their stability at the active site pocket and their unique structure will make them insusceptible to β-lactamase activity.

Furthermore, the dual-site inhibition strategy, targeting both the allosteric and active sites, offers a significant advantage over single-site approaches. This mechanism not only disrupts the enzymatic function of PBP2a more effectively but also mitigates the impact of mutations that confer resistance. This mechanism aligns with recent structural insights into PBP2a allosteric regulation [13]. The favorable *in silico* ADMET profiles of these compounds, particularly Dichamanetin’s high gastrointestinal absorption, further support their candidacy for preclinical development. Although Uvarinol’s bioavailability may require optimization through formulation strategies (e.g., nanoparticle encapsulation or prodrug development, among others), its potent binding energy and interaction profile make it a promising template for designing novel antibiotics. Notably, previous studies have demonstrated that Dichamanetin exhibits potent antimicrobial activity against Gram-positive bacteria [38], including MRSA [39], with MIC and MBC of 2 and 7.5 µg/mL, respectively. The values were better and very promising when compared with the standard antibiotics used: amoxicillin (MIC  =  62 and >250 µg/mL, respectively). This supports the plausibility of our *in silico* predictions.

Overall, these phytochemicals represent a promising alternative to conventional β-lactam antibiotics. Their dual-site binding capability, robust interaction with key residues, and complementary binding strategies could be harnessed either as stand-alone therapeutics or in synergy with existing antibiotics to combat MRSA.

Previous phytochemical analyses of *U. chamae* roots and leaves have reported these compounds as present in appreciable amounts, particularly in ethanolic extracts [23,26]. Quantitative extraction studies indicate yields ranging from 2.03% to 9.8% (*w*/*w*) for conventional and ultrasound-assisted hydroalcoholic extraction, respectively [40]. This yield is relatively high compared to other natural sources of flavonoids and supports their scalability for further drug development efforts. Future in vitro and in vivo studies are essential to validate these computational findings and further explore the clinical potential of Uvarinol and Dichamanetin.

## 4. Materials and Methods

### 4.1. Virtual Screening and Docking Platform

Phytochemicals that have been previously reported to be isolated from *U. chamae* were retrieved from the PubChem online database and used for this study. The Schrödinger Suite software, Maestro 11.5 was used for protein and ligand preparations, while AutoDock tools were used for the molecular docking study, following the standard molecular docking principle [41,42].

### 4.2. Phytochemical Library Generation and Ligand Preparation

The two-dimensional (2D) structures of phytochemicals from the *U. chamae* plant in SDF format were retrieved from the PubChem online database; https://pubchem.ncbi.nlm.nih.gov/ (accessed on 29 September 2023). The phytochemicals (123 compounds) were obtained from research reviews of the plant [24]. The ligprep tool of the Schrödinger suite’s Maestro was used to transform the 2D structures into 3D structures by the addition of hydrogen atoms, ionization at pH (7.2 ± 0.2), and removal of salt using Ep2i/UNEP/-Zk. The ionization and tautomeric state formation was performed using the OPLS3e force field.

### 4.3. Target Retrieval and Preparation

The X-ray crystal structure of the PBP2a proteins, with bound ligands at the allosteric site (PDB ID: 4CJN, Resolution: 1.95 Å) [15], and the active site (PDB ID: 1MWU, Resolution: 2.60 Å) [10], were retrieved from the Protein Data Bank (https://www.rcsb.org, accessed on 18 December 2024). PyMOL Molecular Graphics System, Version 2.5 Schrödinger, LLC (New York, NY, USA) was used for visualization of the proteins. The proteins were prepared using the protein preparation wizard tool in Maestro’s Schrodinger Suite. Following standard protocols, bond orders were assigned, hydrogens added, zero-order metal bonds made, disulfide bonds created, water molecules removed, and het states were generated using Epik at pH 7.0 ± 0.2 during the protein preparation process. The protein refinement was performed by optimizing the H-bond assignment, and then the protein was reduced using the OPLS3e (optimized potentials for liquid simulation) force field.

### 4.4. Receptor Grid Generation

The receptor grid depicts the area where the ligand and protein interact. The coordinate of the co-crystallized ligand was used to specify and generate the receptor grid/active site for docking on AutoDock. By selecting the co-crystallized ligand at the active site of the receptor, the binding location was automatically mapped (by a cubic grid box) covering all of the amino acid residues at the active site. The grid’s three-dimensional coordinates are X= 8.95 Å, Y= −1.38 Å, and Z= −69.93 Å for 4CJN and X= −36.62 Å, Y= 47.0 Å, and Z= 66.24 Å for 1MWU, respectively.

### 4.5. Molecular Docking

Molecular docking was performed using AutoDock tools (AutoDock 4) to assess the binding interactions of the selected phytochemicals from *Uvaria chamae* at the active and allosteric sites of PBP2a. The docked poses were ranked based on their binding energies, and the best-ranked pose for each ligand was selected based on the lowest binding energy and key molecular interactions. To validate the docking protocol, the co-crystallized ligands were re-docked into their respective binding sites, and the root mean square deviation (RMSD) between the re-docked and original ligand poses was calculated. An RMSD value below 2.0 Å was considered indicative of reliable docking. The 2D protein-ligand interaction diagrams were generated using the 2DPoseView option of the ProteinPlus (http://proteins.plus, accessed on 18 December 2024) web server and visualization was performed using the PyMOL molecular graphics system (version 2.5, Schrödinger, LLC., New York, NY, USA).

### 4.6. Pharmacokinetic Profile

The in silico pharmacokinetic properties of the selected phytochemicals, including absorption, distribution, metabolism, excretion, and toxicity (ADMET), were predicted using the SwissADME web tool (http://www.swissadme.ch/, accessed on 18 December 2024) [43]. Canonical SMILES of each compound were input into SwissADME, and parameters such as Lipinski’s Rule of Five, water solubility, gastrointestinal (GI) absorption, blood–brain barrier (BBB) permeability, and drug-likeness were evaluated. Metabolic stability was predicted by assessing interactions with cytochrome P450 (CYP) enzymes, specifically CYP1A2, CYP2C19, CYP2C9, CYP2D6, and CYP3A4. The synthetic accessibility of the compounds was also estimated to determine the feasibility of chemical synthesis. Additionally, the bioavailability score was calculated to predict oral absorption potential. The combined ADMET analysis provided a comprehensive overview of the drug-like properties of Uvarinol and Dichamanetin, aiding in their potential as lead compounds for further development.

### 4.7. Molecular Dynamic (MD) Simulation and Trajectory Analysis

The Desmond module of the Schrödinger software was utilized to perform the Molecular Dynamic (MD) simulation for the native PBP2a (4CJN) and the two complexes (4CJN-Dichamanetin and 4CJN-Uvarinol). The system setup, MD production, and trajectory analysis were performed in a similar way, as we have previously reported [30]. All simulations were carried out using the OPLS3e force field. The complexes were bound in an orthorhombic box, with the box size calculation method set as buffer, all three distances set at 10 Å, then the volume of the box minimized. The TIP3P water model was used as the solvent model. Sodium and chloride ions were added to neutralize the overall charge of the system, and the salt concentration was set to 0.15M, to mimic physiological conditions. The standard protocols within the Maestro environment were employed to prepare and minimize the system. The system relaxation was performed in an NPT ensemble at 300 K and 1 atm, using a Nose-Hoover thermostat and a Martyna-Tobias–Klein barostat, respectively. The production MD simulation was performed for 100 nanoseconds (ns), and the trajectory sampling was set at an interval of 100 ps with 1000 frame numbers, allowing for extensive sampling of the conformational space. During the MD simulation, the long-range electrostatic interactions were calculated by using the Particle Mesh Ewald (PME) method. The outputs of the simulation were visualized and analyzed by an MS-MD trajectory analysis and a simulation interaction diagram. The data were plotted using Origin version 6.0.

### 4.8. Statistical Analysis

The statistical analysis—means and standard error of means (SEM) of the interactive properties from the MD simulation and generation of the heatmap (Figure 1)—were performed using GraphPad Prism version 9.0.0 for Windows, GraphPad Software, San Diego, CA, USA (www.graphpad.com, accessed on 18 December 2024).

## 5. Conclusions

This study demonstrated that phytochemicals from *Uvaria chamae*, notably Uvarinol and Dichamanetin, hold significant promise as dual-site inhibitors of PBP2a, a critical enzyme implicated in MRSA resistance. By simultaneously engaging both the active and allosteric sites of PBP2a, these compounds would disrupt the enzyme’s function more effectively than conventional inhibitors. Binding free energy analyses indicate that both Uvarinol and Dichamanetin form stable, energetically favorable complexes with PBP2a, with Uvarinol exhibiting a particularly robust binding profile and Dichamanetin achieving stability through a compact binding mode. Furthermore, their distinct structural attributes and favorable ADMET profiles, especially that of Dichamanetin, underscore their potential as templates for novel antimicrobial agents. These findings pave the way for further in vitro and in vivo validation, ultimately contributing to the development of alternative therapeutic strategies to combat MRSA and other resistant pathogens.

## Figures and Tables

**Table 1:** Shows the docking scores (Kcal/mol), predicted inhibition constant (Ki) and the interacting residues of the allosteric site (4CJN) and active site (1MWU) with their co-crystallized ligands and best hit compounds.

Target	Compound	Docking Score	Inhibition Constant (Ki)	Key Interaction
**4CJN**	CCL (QNZ)	−9.16	192.16 nM	**H-bond:** LYS273, LYS316 **H-phobic:** ASN104, ASN146, TYR297 **Pi-pi:** TYR105, TYR297
	Dichamanetin	−10.32	27.07 nM	**H-bond:** LYS316 **H-phobic:** ASN104, TYR105, ILE144, LYS273, TYR297 **Pi-pi:** TYR105, TYR297
	Uvarinol	−10.42	22.89 nM	**H-bond:** LYS316, TYR105 **H-phobic:** ASN104, TYR105, GLU154, ASN146, TYR297 **Pi-pi:** TYR297
	Chamuvaritin	−9.17	189.39 nM	**H-phobic:** ASN104, TYR105, ASN146, TYR297, ILE309 **Pi-pi:** TYR105, TYR297
**1MWU**	CCL (7EP)	−8.49	601.23 nM	**H-bond:** SER403, SER462, ASN464, GLN 521, ALA601 **H-phobic:** ILE614
	Dichamanetin	−12.01	1.57 nM	**H-bond:** SER403, TYR 446, SER462, ASN464, LYS606 **H-phobic:** GLY402, TYR446, GLY611, ILE614, TRP616
	Uvarinol	−14.94	0.01 nM	**H-bond:** SER400, SER403, SER462, ASN464, GLN 521, GLY522, ARG612, GLN613, ILE614 **H-phobic:** TRY446, GLN521, GLU602
	Chamuvaritin	−11.25	5.66 nM	**H-bond:** SER403, THR444, ASN464 **H-phobic:** THR600, GLU602, LYS606, ILE614

Note: CCL—Co-crystallized Ligand; H-bind—Hydrogen bond; H-Phobic—Hydrophobic.

**Table 2:** Interactive properties of MDs of the ligands and the PBP2a allosteric (4CJN) and active (1MWU) sites.

Receptor	Ligand	P_RMSF	PL_RMSD	rGyr	MolSA	SASA	PSA
4CJN	Quinazolinone	2.182 ± 0.043	4.083 ± 0.024	4.478 ± 0.002	362.8 ± 0.09	291.2 ± 1.92	179.8 ± 0.11
	Dichamanetin	1.674 ± 0.027	4.752 ± 0.018	4.312 ± 0.002	400.9 ± 0.12	224.2 ± 0.94	124.1 ± 0.14
	Uvarinol	1.956 ± 0.040	3.386 ± 0.016	5.452 ± 0.002	496.6 ± 0.13	444.4 ± 1.172	164.8 ± 0.18
	Ceftaroline	1.810 ± 0.033	2.625 ± 0.015	6.147 ± 0.011	533.3 ± 0.26	355.9 ± 1.602	371.1 ± 0.25
1MWU	7EP (CCL)	1.809 ± 0.030	4.448 ± 0.027	3.859 ± 0.002	347.1 ± 0.11	447.8 ± 7.417	181.8 ± 0.20
	Dichamanetin	2.139 ± 0.039	3.036 ± 0.021	4.303 ± 0.002	402.1 ± 0.15	213.9 ± 7.967	142.5 ± 0.32
	Uvarinol	2.052 ± 0.039	2.955 ± 0.022	5.294 ± 0.006	499.6 ± 0.13	294.9 ± 8.826	170.1 ± 0.21
	Ceftaroline	2.030 ± 0.040	3.550 ± 0.023	6.450 ± 0.004	541.6 ± 0.13	195.0 ± 1.134	337.6 ± 0.32

Note: Values are presented as means ± standard error of mean (SEM) measured in Armstrong units (Å). PL_RMSD: Complex root mean square deviation; P_RMSF: protein root mean square fluctuation; rGyr: radius of gyration; MolSA: molecular surface area; SASA: solvent-accessibility surface area; PSA: polar surface area.

**Table 3:** MM/GBSA values of 4CJN and 1MWU protein–ligand complex calculated using the MD trajectories.

Receptor	Ligand	MM/GBSA
4CJN	Quinazolinone	−37.25 ± 0.76
	Dichamanetin	−43.48 ± 0.50
	Uvarinol	−44.30 ± 0.42
	Ceftaroline	−65.82 ± 0.81
1MWU	7EP (CCL)	−48.31 ± 0.87
	Dichamanetin	−54.01 ± 1.35
	Uvarinol	−70.82 ± 0.87
	Ceftaroline	−103.2 ± 0.67

Values are presented as mean ± standard error of mean (SEM) measured in kcal/mol.

**Table 4:** In silico drug-likeness prediction of Quinazolinone, Dichamanetin, and Uvarinol.

Compound	MW	#H-Bond Acceptors	#H-Bond Donors	TPSA	Consensus Log P	#Lipinski Violation	Synthetic Accessibility
Quinazolinone	393.39	5	1	95.98	3.61	0	3.10
7EP	382.43	7	3	139.26	0.05	0	4.01
Ceftaroline	684.68	12	4	340.13	−1.16	2	5.75
Dichamanetin	468.50	6	4	107.22	4.44	0	4.15
Uvarinol	574.62	7	5	127.45	5.63	1	4.70

**Table 5:** The predictive pharmacokinetic properties of Quinazolinone, Dichamanetin, and Uvarinol.

	GI Absorption	BBB Permeant	P-Glycoprotein Substrate	CYP1A2 Inhibitor	CYP2C19 Inhibitor	CYP2C9 Inhibitor	CYP2D6 Inhibitor	CYP3A4 Inhibitor	Bioavailability Score
Quinazolinone	High	No	No	No	No	Yes	No	No	0.56
7EP	High	No	Yes	No	No	No	No	No	0.55
Ceftaroline	Low	No	Yes	No	No	No	No	No	0.11
Dichamanetin	High	No	No	No	Yes	Yes	No	No	0.55
Uvarinol	Low	No	No	No	No	No	No	No	0.55

## Data Availability

The data presented in this study are available upon request from corresponding authors.
